# Host-Parasite Interactions from the Inside: Plant Reproductive Ontogeny Drives Specialization in Parasitic Insects

**DOI:** 10.1371/journal.pone.0139634

**Published:** 2015-10-06

**Authors:** Thomas Boivin, Cindy Gidoin, Patrick von Aderkas, Jonathan Safrana, Jean-Noël Candau, Alain Chalon, Marion Sondo, Mohamed El Maâtaoui

**Affiliations:** 1 INRA, UR 629 Ecologie des forêts méditerranéennes, F-84914, Avignon, France; 2 University of Victoria, Department of Biology, Centre for Forest Biology, Victoria, BC, V8W 3N5, Canada; 3 Natural Resources Canada, Canadian Forest Service, Great Lakes Forestry Centre, Sault Ste Marie, ON, P6A 2E5, Canada; 4 Univ Avignon, EA 4279, BP 21239, F-84916, Avignon, Cedex 9, France; University of Basilicata, ITALY

## Abstract

Host plant interactions are likely key drivers of evolutionary processes involved in the diversification of phytophagous insects. Granivory has received substantial attention for its crucial role in shaping the interaction between plants and their seed parasites, but fine-scale mechanisms explaining the role of host plant reproductive biology on specialization of seed parasites remain poorly described. In a comparative approach using plant histological techniques, we tested the hypotheses that different seed parasite species synchronize their life cycles to specific stages in seed development, and that the stage they target depends on major differences in seed development programs. In a pinaceous system, seed storage products are initiated before ovule fertilization and the wasps target the ovule’s nucellus during megagametogenesis, a stage at which larvae may benefit from the by-products derived from both secreting cells and dying nucellar cells. In a cupressaceous system, oviposition activity peaks later, during embryogenesis, and the wasps target the ovule’s megagametophyte where larvae may benefit from cell disintegration during embryogenesis. Our cytohistological approach shows for the first time how, despite divergent oviposition targets, different parasite species share a common strategy that consists of first competing for nutrients with developing plant structures, and then consuming these developed structures to complete their development. Our results support the prediction that seed developmental program is an axis for specialization in seed parasites, and that it could be an important parameter in models of their ecological and taxonomic divergence. This study provides the basis for further investigating the possibility of the link between plant ontogeny and pre-dispersal seed parasitism.

## Introduction

Seeds are highly sought after by many animal species as they are extremely aggregated in space with high local abundance relative to other food resources, and as they generally contain nutrient with greater energy than other plant structures [1]. Granivory has received substantial attention for its crucial role in plant demography, spatial distribution, diversity and evolution [2–4]. Moreover, there are numerous features that distinguish granivory from other forms of herbivory in shaping the interaction between plants and their seed parasites [5,6]. Host plant interactions are well-established drivers of the evolutionary processes involved in the diversification of phytophagous insects [7–9].

During seed development, most plants invest substantial resources to provide their embryos with compounds upon which they may draw during embryogenesis and germination [10]. To take advantage of this situation many seed parasites evolved towards an endophytic life-cycle within seed tissues during their early development (pre-dispersal seed parasites). The onset of such host-parasite interactions is thus most likely linked with host plant reproductive biology. The two major groups of seed plants, angiosperms and gymnosperms, indeed initiate the allocation of nutrients to the ovule in two different ways. Most angiosperms and some gymnosperms (e.g. Cupressaceae), require pollination to ensure the normal development of both female gametophyte (megagametophyte) and ovule; nutrient products only begin to accumulate following fertilization [11–13]. In some other gymnosperms (e.g. cycads and Pinaceae), storage may begin in megagametophytes prior to fertilization [14], sometimes even in the absence of pollination (e.g. *Pseudotsuga*). However, unpollinated megagametophytes will eventually abort, resulting in a so-called empty seeds [12,15,16]. Such variation in host reproductive pathways presents different targets for seed parasites seeking storage reserves, for which phenological synchrony with the reproductive stages of their host may be under strong selection and drive ecological specialization [6,17,18]. When feeding on seeds of angiosperms or cupressaceous gymnosperms, the parasite’s reproductive success should depend first on fertilization success and then on the ability to find fertilized ovules to lay eggs. Conversely, seed parasites exploiting ovules of pinaceous gymnosperms that produce nutrients even when pollination has not occurred can theoretically target earlier developmental stage of ovules, including pre-fertilization stages [16,19]. Understanding how host-seed parasite interactions take place requires within-season studies of seed parasitism patterns and these have received little attention so far. A major limit to what is currently known is partly a consequence of the tradition of linking phenology of oviposition to external fruit, cone or seed morphology only [20–23]. Plant histological techniques have proven to be successful in investigating the biology and broader evolutionary patterns in cynipid gall wasps [24,25], another family of plant host parasites that share phenological constraints on oviposition with seed endoparasites.

This study aimed at providing cytohistological insights into the role of host reproductive ontogeny in driving seed insect specialization through oviposition strategies. We specifically tested the hypothesis that major differences in seed development programs result in different specific oviposition targets to which seed parasites synchronize their life cycles. Plant parasitism by seed chalcid wasps of the *Megastigmus* genus (Hymenoptera: Torymidae) is an ideal model system with which to address this issue, because this insect group not only occurs on a wide range of angiosperm (Rosaceae and Anarcadiaceae) and gymnosperm hosts (Pinaceae, Taxodiaceae and Cupressaceae), but features strong plant-parasite specificities, which have greatly influenced their evolutionary history [26,27]. *Megastigmus* is a highly invasive genus of over 125 species worldwide, half of which are specialist seed feeders that complete their development within the confines of the seed, at a density of only one larva per seed [28,29]. All species are univoltine and share a similar life cycle. Adults emerge from seeds for a brief period, females oviposit in multiple host trees during a narrow window of development of the host female reproductive structures, and larvae consume both the megagametophyte and the seed embryo before entering a period of developmental arrest (larval diapause) for one to five years [28]. Some *Megastigmus* species target conifer species with ovules at prefertilization stages, others target conifers with ovules that are post-fertilization [16]. In the best-studied system, *M*. *spermotrophus* females can target Douglas-fir ovules with developing megagametophytes within ovules that are at a prefertilization stage [19]. One of the unusual aspects of this host-parasite interaction is that the insect is able to redirect seed ontogeny to the larva’s advantage. Although Douglas-fir megagametophytes are able to develop in the absence of pollen until the time of fertilization, megagametophyte degenerate if ovules are not pollinated. However, if they are parasitized by *M*. *spermotrophus*, unfertilized ovules not only fail to abort, but they begin to accumulate storage reserves as if they had been fertilized. The fattening seed is consumed by the manipulative parasitic larva [19]. A comparative review of published phenologies of cupressaceous and pinaceous conifers suggested that closer investigation of oviposition in relation to ovule developmental stage should reveal more strategies than are currently known [16]. To address this knowledge gap, we tested here whether *Megastigmus* species specialized on Pinaceae differ in the ovular tissues that they target during oviposition from species specialized on Cupressaceae. The pinaceous representative that we studied was the Atlas cedar *Cedrus atlantica* Manetti, which is parasitized by two species, *M*. *schimitscheki* Novitzky and *M*. *pinsapinis* Hoffmeyer. The cupressaceous representative was the evergreen cypress *Cupressus sempervirens* L., which is parasitized by *M*. *wachtli* Seitner. There is phylogenetic evidence of a taxonomic radiation following initial host adaptation between wasp species exploiting pinaceous and cupressaceous hosts [26,27].

In both pinaceous and cupressaceous host-parasite systems, we superimposed the phenologies of wasp oviposition and host reproductive development. By sectioning ovules before, during and after fertilization, we were able to pinpoint the earliest stages of oviposition and identify the timing of seed development as one important factor that could drive the evolution of the specificity of the interactions between plants and their seed parasites.

## Material and Methods

### Study systems

The reproductive development of conifer megagametophytes is conservative. The megaspore mother cells undergo meiotic divisions that result in the production of a tetranucleate coenomegaspore that initiate the megagametophyte. Successive waves of mitosis lead to the formation of a free-nuclear stage. In *Cupressus sempervirens* early megagametophyte development occurs from March until early May [30]. The coenocyte then cellularizes, differentiates archegonia, which mature to form eggs that are receptive early in July. Fertilization is immediately followed by proembryogenesis that extends until the ovule development is suspended in October for winter dormancy [31]. The cypress seed wasp *M*. *wachtli* is a common species specialized on *C*. *sempervirens* occurring from the eastern Mediterranean basin to Southern Europe with records extending back from several centuries [32]. Adults emerge in the beginning of summer from two-year old cones and then oviposit immediately in the young growing ones. The last larval instar enters diapause in late summer of the same year. From studies of *Cedrus deodara* [33], we know that *Cedrus* spp. share the same megagametophyte developmental sequence with *Cupressus*. However, in the pinaceous representative in our study—*Cedrus atlantica*—our knowledge of reproductive phenology is poor as it relies exclusively on the external morphology of the cones [34,35]. Two cedar seed wasps, *M*. *schimitscheki* and *M*. *pinsapinis*, are specialized on the *Cedrus* genus [36,37]. They exploit and compete for *C*. *atlantica* seeds in southern France [38]. *M*. *schimitscheki* and *M*. *pinsapinis* adults emerge in spring (May-June) from seeds released by two-year cones during the previous fall, and then immediately oviposit in young growing cones. Adults of *M*. *schimitscheki* always emerge 10–20 days earlier than *M*. *pinsapinis* ones, while both species enter larval diapause in late August [39].

### Host sampling and cytohistology

Young growing cones were collected in 2011 from trees in two southeastern host populations in which preliminary field work confirmed the presence of their respective seed-specialized wasps: (i) a windbreak plantation of *C*. *sempervirens* in Montfavet, France (43°55'03.90"N, 4°52'46.98"E, 23 m asl.) and (ii) a natural stand of *Cedrus atlantica* in Luberon, France (43°47'47.50"N, 5°14'28.50"E, 670 m asl.). In each host population, five cones per tree were collected weekly and the same day from five trees during the period of oviposition activity of the wasps. This resulted in 11 (from June 22 to August 31) and 10 (from April 6 to June 8) sampling sessions on the same trees for *Cupressus sempervirens* and *Cedrus atlantica*, respectively. Harvested trees were chosen with similar age, size, and exposure to both light and wind. Collecting cones on the same day and on similar trees limits interindividual discrepancies in cone developmental stages in conifers such as Douglas fir, cedar and the evergreen cypress [19,33,40]. All the sites in this study are located in nationally owned stands and as such did not require a specific permission for cone collection for experimental purposes. In addition, this study was formerly approved by the French Ministry of Agriculture, Food and Forests (MAAP) as a contribution to the sanitary characterization of French forest reproductive material. None of the field surveys in the present study involved endangered or protected species.

All cones were longitudinally sliced the day of their collection using a razor blade and immediately fixed in formalin-acetic acid-alcohol (1/1/8, V/V/V). To promote good penetration of the fixative products specimens were subjected to vacuum for 20 minutes and stored in FAA until required. They were then rinsed 4 hours in tap water and placed in 70% alcohol. Ovules were excised from fixed cone slices, dehydrated in a graded alcohol series (80–100%) and embedded in methacrylate resin (Technovit 7100, Heraeus-Kulzer GmbH, Wehrheim, Germany). Sections (3 μm thickness) were serially cut using a rotary microtome (Reichert-Young Supercut 2065, Wien, Austria), and were then mounted on microscope slides. Periodic acid Schiff’s reagent (PAS) procedure was used to visualize polysaccharides (pink), and Naphthol Blue Black was used to visualize proteins (dark blue) [40]. Images were captured using a Leica DFC 300 FX digital camera mounted on a DMR light microscope and analysed using LAS software (Leica). Among each lot of 25 cones collected the same day, at least three randomly chosen samples were sectioned and then analysed per date of collection. Attention was paid to megagametophyte and embryo development in parasitized ovules.

### Parasite sampling and oviposition timing

The *Cupressus sempervirens* plantation at Montfavet was sampled for diapausing *Megastigmus wachtli* larvae in February 2011. Ten two-year-old cones per tree were collected randomly from each of 30 trees. Cypress cones naturally open and release seeds a few weeks after collection. Seeds were maintained in well-aired, transparent plastic boxes under natural conditions for the duration of the experiment. In November 2010, diapausing *M*. *schimitscheki* larvae were sampled by collecting two-year old cones of *C*. *atlantica* in the Luberon natural cedar stand. *M*. *pinsapinis* larvae were sampled at the same period but in La Vis, another natural cedar stand (Table 1), because this species is strongly outcompeted by *M*. *schimitscheki* at Luberon [38]. This results in too low *M*. *pinsapinis* abundance levels for sampling in this area, although this species can still be observed occasionally in the field. Previous work on cedar wasps showed that French larvae populations collected in different stands but reared in the same conditions after collection were consistently synchronous at adult emergence [39]. For *M*. *pinsapinis*, we therefore expected that the La Vis population could be used as a surrogate of the existing but not sampled Luberon population when it came to examining the question of phenology matching between cedars and *M*. *pinsapinis* reproduction time schedule. In each stand, five two-year-old cones were collected per tree from 10 trees. Cones were individually dissected to extract the seeds. Each of the *C*. *atlantica* seed lots were maintained in well-aired, transparent plastic boxes under natural conditions for the duration of the experiment. Wasp-infested seed lots of *Cupressus sempervirens* and *Cedrus atlantica* were kept and surveyed at the sites where cones were sampled for the cytohistological studies (i.e. Montfavet and Luberon, respectively) to ensure that the observed phenologies of both hosts and parasites resulted from similar local environmental conditions.

**Table 1:** Seasonal timing of female emergence in seed wasps specialized on *Cedrus atlantica* (*M*. *schimitscheki* and *M*. *pinsapinis*) and *Cupressus sempervirens* (*M*. *wachtli*) in southeastern France. Estimates of 50% emergence events and their 95% confidence intervals (95% CI) were calculated using a probit analysis, in which emergence proportions and Julian dates were transformed to probits and log10, respectively.

		Sample collection			Female emergences in 2011		
Host-plant (family)	Wasp species	Sample site	GPS coordinates	Year of collection	N	Temporal range	Emergence 50% (95% CI)
*C*. *atlantica* (Pinaceae)	*M*. *schimitscheki*	Luberon	43°47'47.50"N, 5°14'28.50"E	2010	90	May 2–15	May 9 (May 8–10)
	*M*. *pinsapinis*	La Vis	43°54'27.33"N, 3°29'26.89"E	2010	451	May 12–27	May 18 (May 16–20)
*C*. *sempervirens* (Cupressaceae)	*M*. *wachtli*	Montfavet	43°55'03.90"N, 4°52'46.98"E	2011	178	July 4-August 11	July 20 (July 17–23)

*Megastigmus* female oviposition begins as soon as they emerge. Most of the eggs of a female are laid during the first week post-emergence [16,20,39,41]. This allows us to use the emergence period as a proxy of the oviposition period, and to consider that the time period during which 50% of emergence had occurred coincided with the highest levels of oviposition activity. In each seed lot, wasp adult emergences were recorded daily and each emerging individual was identified to species and sexed (see Tables A and B in S1 Dataset). Preliminary tests for normality of data were performed by using a Kolmogorov-Smirnov test at the 5% threshold. Emergence patterns of females were further expressed as daily cumulative percentages of emergence, and then characterized using their temporal range and their 50% frequency of occurrence estimated using a probit analysis [42,43]. For this purpose, emergence proportions and Julian dates were transformed to probits and log10, respectively. In an iterative approach, an approximate linearity of the relationship between the transformed variables allowed estimations of linear regression parameters, from which the 50% emergence events and their associated 95% confidence intervals were calculated. Probit analyses were performed using the R program [44]. At each site, daily visual observations of the behaviour of wasps were done to check for consistency between adult emergence records from our seed lots and female oviposition activity in the wild. One hour-long direct observations of ovipositing *M*. *wachtli* (at Montfavet) and *M*. *schimitscheki* and *M*. *pinsapinis* (at Luberon) females were done on 30–40 trees located around the seed lots.

## Results

### Parasitism strategy in the cupressaceous system

**Fig 1:**
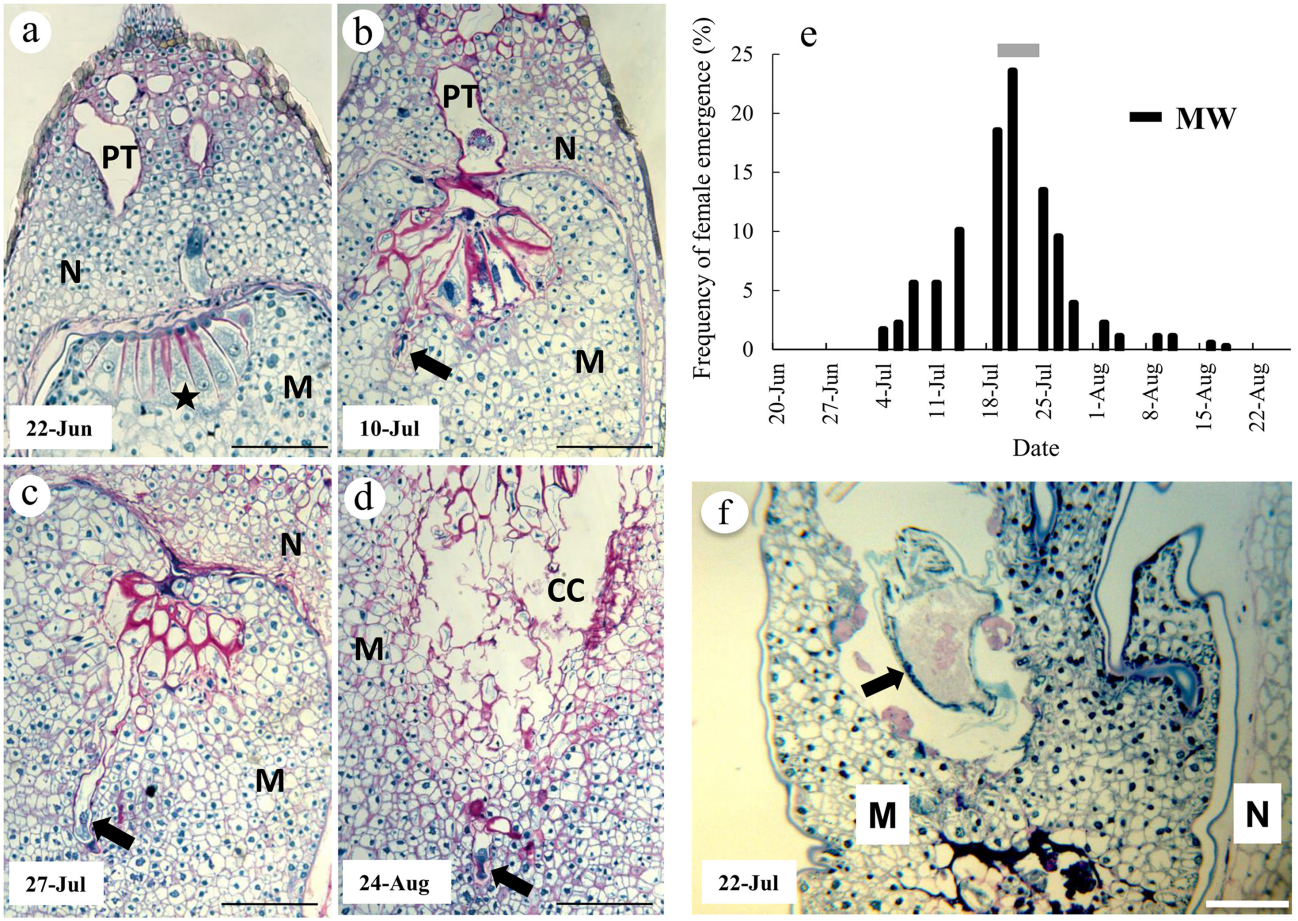
Comparison of the ontogeny of *Cupressus sempervirens* female reproductive structures with seasonal timing of the oviposition activity of the seed wasp *Megastigmus wachtli* (MW) in southeastern France. **a**, Longitudinal section of an ovule collected on June 22, 2011 showing a megagametophyte at prefertilization stage with archegonial complex (star). Note the presence of pollen tubes within the nucellus. **b**, Post-fertilisation megagametophyte from an ovule collected on July 10, 2011. The early proembryo (arrow) is just emerging from the fertilized archegonium. **c**, and **d**, Sections from ovules collected on July 27 and August 24, 2011, respectively showing proembryos (arrows) within the megagametophyte tissues. CC, corrosion cavity; M, megagametophyte; N, nucellus; PT, pollen tubes. All scale bars 200 = μm. **e**, Frequencies of oviposition activity in MW; oviposition begins at female emergence. Grey bars: periods of maximal oviposition activity. **f**, Section of a parasitized *C*. *sempervirens* young seed collected on July 22, 2011 and showing a larva (arrow) within the megagametophyte tissue. M, megagametophyte; N, nucellus. Bar 200 = μm.

We compared the ontogeny of *Cupressus sempervirens* female reproductive structures with seasonal timing of the oviposition activity of the seed wasp *M*. *wachtli* (Fig 1). In the *Cupressus sempervirens* seed lot, adult females of *M*. *wachtli* emerged from July 4 to August 11, 2011, with the highest emergence rates estimated around the July 20 (Table 1; Fig 1e). Daily field observations at Montfavet revealed that *M*. *wachtli* females were ovipositing on cypress cones from July 9 to August 20, 2011, which confirmed that emergence records in our cypress seed lots were a reliable proxy of the seasonal timing of oviposition in the wild. Cones of *C*. *sempervirens* were collected once a week during 11 weeks from June 22 to August 31, 2011, a period that encompassed the wasps’ oviposition periods. This allowed us to describe the reproductive ontogenic stages achieved by cypress ovules before (June 22, 2011), during (July 10 and 27, 2011) and after (August 24, 2011) the highest wasp oviposition-activity period (Fig 1a–1d).

Cytohistological analyses of *C*. *sempervirens* samples showed that the reproductive ontogeny conformed to the pattern reported by Sallandrouze et al. [31]. Ovules collected on June 22, 2011 had cellularized megagametophytes with mature archegonia (Fig 1a). At this prefertilization stage, the female gametophytic tissue constituted the major component of ovules. Except in the micropylar region (Fig 1a) where the nucellus was thicker, the nucellus was generally reduced to few cell layers. Of these, the innermost layers in contact with megagametophyte were in a state of disintegration. Ovules collected on July 10, 2011 were fertilized: proembryos were observed that had begun to enter the surrounding female gametophytic tissue (Fig 1b). Ovules collected on July 27 and on August 24, 2011 had early-stage embryos more fully pushed into the megagametophyte (Fig 1c and 1d). By August 24, 2011, a corrosion cavity had formed by the disintegration of the most central cells of the megagametophyte that surrounded the developing embryos (compare Fig 1c and 1d).

Larvae were observed in sections of parasitized megagametophytic tissues collected on July 22, 2011 (Fig 1f). The larvae were surrounded by remnants of collapsed megagametophyte cells as well as by amorphous materials resulting from cell death.

### Parasitism strategy in the pinaceous system

**Fig 2:**
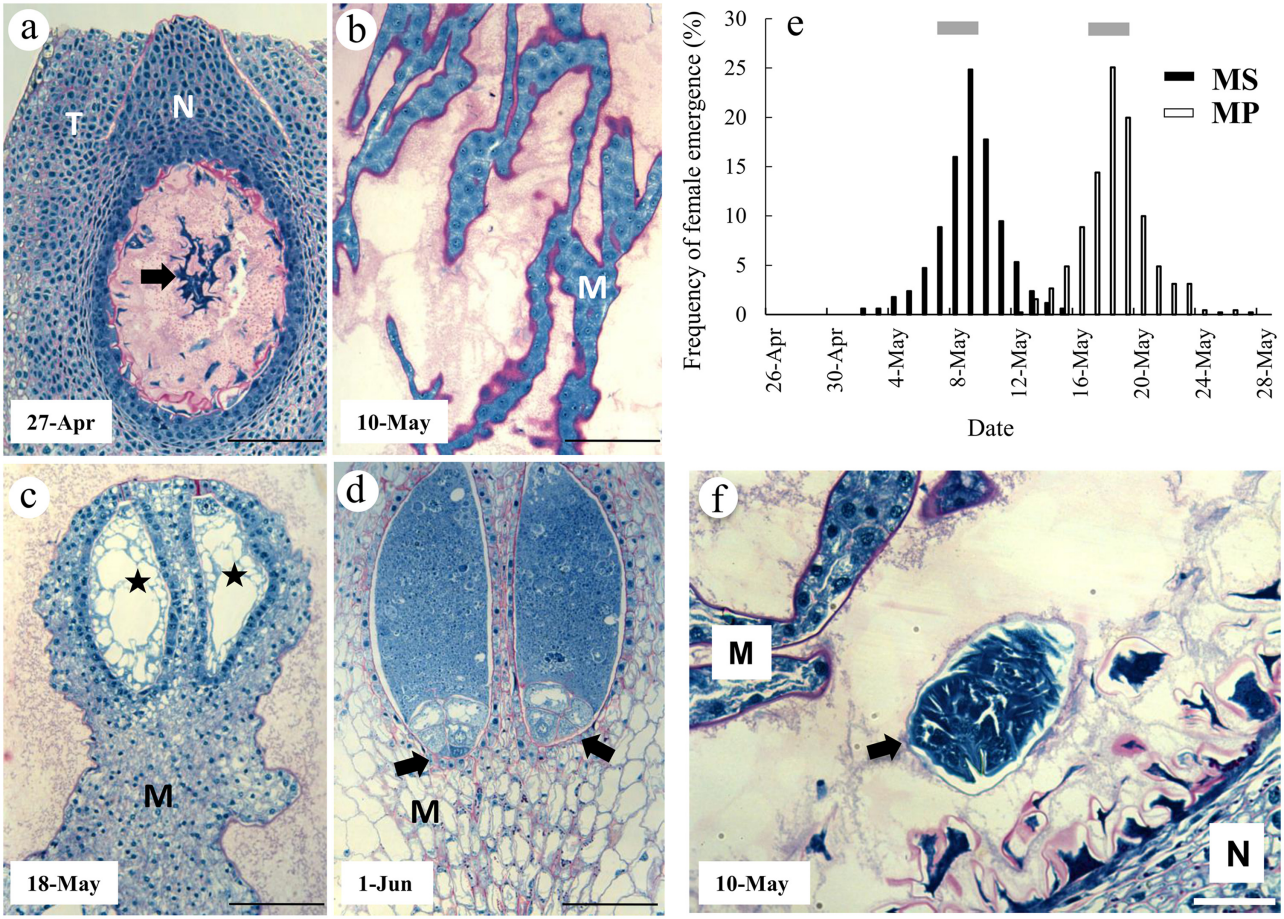
Comparison of the ontogeny of *Cedrus atlantica* female reproductive structures with seasonal timing of the oviposition activity of the seed wasps *M*. *schimitscheki* (MS) and *M*. *pinsapinis* (MP) in southeastern France. **a**, Longitudinal section of an ovule collected on April 27, 2011 showing integuments (T) and nucellus (N). The megagametophyte (arrow) occupies the central cavity within an amorphous, PAS positive matrix derived from the disintegration of nucellar cells. **b**, Megagametophyte at early cellularization stage from an ovule collected on May 10, 2011. Note the presence of PAS positive substance all around the megagametophyte extensions. **c**, Section from an ovule collected on May 18, 2011 showing cellularized megagametophyte with early archegonia initials (stars). **d**, Section from an ovule collected on June 1, 2011 showing fertilized archegonia at early proembryogenesis (arrows). M, megagametophyte; N, nucellus; T, integuments. Scale bars 150 = μm, **a** and **c**; 75 μm, **b** and **d**. **e**, Frequencies of oviposition activity in MS and MP; oviposition begins at female emergence. Grey bars: periods of maximal oviposition activity. **f**, Section of a *C*. *atlantica* parasitized ovule collected on May 10, 2011 and showing a larva (arrow) between the degenerating nucellus (N) and the cellularized megagametophyte (M). Bar 200 = μm.

We compared the ontogeny of *Cedrus atlantica* female reproductive structures with seasonal timing of the oviposition activity of the seed wasps *M*. *schimitscheki and M*. *pinsapinis* (Fig 2). As expected [39], adult females of *M*. *schimitscheki* emerged earlier (from May 2 to May 15, 2011) than those of *M*. *pinsapinis* (from May 12 to May 27, 2011) in the *C*. *atlantica* seed lots. The highest emergence rates for *M*. *schimitscheki* and *M*. *pinsapinis* were estimated to be around the May 9 and May 18, 2011, respectively (Table 1; Fig 2e). This reflected the typical phenological discrepancy previously described for these species [39]. Complementary daily field observations revealed that *M*. *schimitscheki* and *M*. *pinsapinis* females were ovipositing on Luberon cedar cones from May 5 to May 28, 2011 and from May 9 to June 5, 2011, respectively. These results confirmed that emergence records in our cedar seed lots were a reliable proxy of the seasonal timing of oviposition in the wild. Cones of *C*. *atlantica* were collected once a week during 10 weeks from April 6 to June 8, 2011, which clearly encompassed the wasps’ oviposition periods. This allowed us to describe the reproductive ontogenic stages of cedar ovules before the highest wasp oviposition activity period (April 27, 2011), as well as during (May 10 and May 18, 2011) and after this period (June 1, 2011) (Fig 2a–2d).

Cytohistological analyses of *C*. *atlantica* ovules sampled on April 27, 2011 showed that the megagametophytes were at an early free-nuclear phase (Fig 2a). They had a protein-rich cytoplasm that occupied the central region of the ovules. The surrounding nucellar cells appeared to be subjected to an intense lysis as attested by the presence of numerous profiles of collapsing cells with shrunken cytoplasm and disintegrating walls (Fig 2a). A mucilaginous zone composed of a mixture of polysaccharides and proteins is found surrounding the megagametophyte. Sections from ovules harvested on May 10, 2011 had well developed megagametophytes at an early stage of cellularization (Fig 2b). At this stage, nucellar cell breakdown produced a large cavity (not shown) containing disintegrating cells and a mucilage-like substances staining positive for polysaccharides and proteins (Fig 2b). Ovules collected on May 18, 2011 had megagametophytes undergoing archegonial formation (Fig 2c). Fertilization had occurred by June 1, 2011: proembryos were observed in corrosion cavities (Fig 2d). The megagametophyte tissue occupied almost the entire volume of central ovule; of the nucellus, only a thin layer of mucilaginous material remained (not shown).

Larvae were found within the disintegration zone of the innermost nucellar tissue of parasitized ovules collected on May 10, 2011 (Fig 2f). Larvae were surrounded by cell wall remnants of collapsing nucellar cells and by detritus due to cell disintegration. What differed in the ovular tissues between parasitized and non-parasitized seeds was only the presence of a larva and tissues that have been consumed. We did not detect any particular specialized structure resulting from insect parasitism.

## Discussion

A central finding of our study is the implication that ecological specialization in pre-dispersal seed parasites is possibly due to synchronization of oviposition timing with the onset of nutrient formation within the targeted plant tissues. In the life cycle of coniferous hosts both megagametogenesis and embryogenesis represent nutrient-abundant stages [12], our data show that early stages of larval development in *Megastigmus* species have specialized to take advantage of these situations to the detriment of seed development. Two different nutrient sources for *Megastigmus* larvae are characterized in our Pinaceae-Cupressaceae comparative approach. In the pinaceous system, oviposition activity in both *M*. *schimitscheki* and *M*. *pinsapinis* peaks during megagametogenesis. At this stage, the cytohistological analysis shows that inner nucellar cells are subjected to an intense lytic process, similar to programmed cell death, which results in the production of an amorphous, mucilage-like substance rich in both polysaccharides and proteins. Other studies have shown that sporopollenin may be released from these tapetum-like layers, providing materials to the developing megaspore wall [45]. Interestingly, in all the parasitized ovules examined at this stage, eggs and early larvae are observed to be confined to the inner nucellus, precisely in the vicinity of collapsing cell layers. This suggests that the primary resources targeted by the cedar wasp larvae are the by-products derived from secreting cells as well as from dying nucellar cells. In agreement with previous research on Douglas fir and *M*. *spermotrophus* [19], we speculate that parasite targeting of early ovule development followed by larva’s redirection of host seed ontogeny is common on the Pinaceae. In the cupressaceous system, oviposition activity in *M*. *wachtli* peaks later, during embryogenesis. By this stage, the ovule’s nucellus has largely disappeared. As a result, larvae are confined to the interior of the megagametophyte, where they probably benefit from both collapsing cells and the amorphous material generated by cell disintegration during corrosion cavity formation that accompanies embryogenesis. These results are consistent with similar approaches conducted on gall wasp communities (Hymenoptera: Cynipidae), which also require specific tissues at specific developmental stages in their hosts, and for which the timing and site of oviposition are essential to successful gall development [46,47]. The precise deposition of eggs in leaf bud tissues is key to species specific differences in gall structure, and may have influenced species radiation in the single genus *Diplolepis* on wild roses [25]. However, we do not detect any wound response to the presence of a larva in both cedar and cypress ovules, i.e. a layer of tissue formed in response to larval feeding activity, as would be found in galls. This implies that these seed wasps evolved towards the use of a plant structure that is naturally acting as a sink for nutrients. Apparently tolerated by the plant, larval development is then favoured by oviposition in this sink. Post-fertilization *Megastigmus* species thus contrast with gall wasps by apparently exploiting their host plant passively at the appropriate time in development, instead of initiating its developmental program as gallers do. Whether pre-fertilization *Megastigmus* species may be considered as gallers by redirecting ovule development is still a matter of debate because the insect does not induce a novel plant structure from a histological point of view, but the larva still prevents megagametophyte degeneration and induces differentiation of plant storage tissues [19]. The mechanisms involved in the prevention of seed abortion are still unknown. One could however postulate that the larva maintains the gradient of the nutrient sink by its rate of consumption of plant tissue, which possibly define a new guild in insect-plant interactions. Comparing such phytophagous insect groups that evolved towards intimate relationships between host phenology and resource supply to larvae, we posit that the strength of host-parasite interaction ranges from passive host exploitation (post-fertilization seed wasps), to active host manipulation without new host structure (pre-fertilization seed wasps) and to active host manipulation with new host structure (gall wasps).

Host plant interactions have been considered for a long time as critical drivers of the evolutionary processes involved in the diversification of phytophagous insects [7–9]. The specificity of the interactions between plants and their parasites depends on a number factors, one of which is trophic mode. Previous research has indicated that pre-dispersal seed parasites tend to be exceptionally host specific [26,27,48,49]. Parasitism during the seed pre-dispersal phase provides critical study cases in this context as it tends to exclude generalists, while favouring specialists. Insects are indeed adapted to synchronize their life-cycles with the ephemeral availability of seeds from a few closely related host plants, which is likely to reflect specialization [6]. In particular, insect species in which both oviposition and larval development occur on immature seeds may be under strong selection for synchronizing their life history with appropriate reproductive stages of their host plant [17,18]. By linking both the phenologies and the targets of oviposition in highly-specialized seed wasps and the reproductive ontogeny of their hosts, we provide here novel cytohistological appreciation of how specific host-seed parasite interactions may be shaped.

Cones of conifers generally display continuous development in both size and internal structure that leads to a progressive lignification following embryogenesis and during the seed maturation phase [28,50]. Thus, even if seed wasps have specialized through the exploitation of different nutrient sources at the time eggs are laid within the ovule, oviposition success should be primarily linked to penetration success through the cone scale and bract complex. In the Pinaceae, intensive cone structural changes with time define a narrow favourable window for *Megastigmus* to oviposit as females lay eggs by inserting their ovipositor directly through the young cone scales [16]. Their ability to access ovules thus decreases as the scales harden or their size exceeds that of the ovipositor [38,39]. Conversely, temporal cone structural changes in the Cupressaceae may not severely affect female penetration success as the size of a mature cone is not a limiting factor with respect to ovipositor length, and *Megastigmus* females tend to penetrate between or at the external limit of cone scales, which is likely to limit physical constraints to oviposition [21,32]. Consequently, parasite specialization on the Pinaceae may also result from selection for synchronizing oviposition timing with early morphological stages of cones, while specialization on the Cupressaceae may have been shaped independently from temporal changes in cone structure. This is supported by the shorter adult emergence spread in cedar wasps (2–3 weeks) than in the cypress wasp (6 weeks).

Animals are expected to develop foraging strategies that maximize fitness, e.g. many herbivore insect species have adjusted their phenology and oviposition choices with the occurrence of target host organs that maximize growth and survival of their progeny [51,52]. Our work suggests that both pre- and post-fertilization parasitism strategies might have noticeably divergent fitness implications for seed-specialized parasites. In the case of post-fertilization seed parasitism, this study provides the first evidence that parasites synchronize oviposition timing with the beginning of embryogenesis, i.e. when the storage reserve accumulation that is essential to larval development is triggered. Our approach suggests that a similar strategy may be used in angiosperm seed parasites, such as *Macrodasyceras hirsutum* Kamijo (Hymenoptera: Torymidae) which oviposit exclusively in fertilized seeds of the mochi tree, *Ilex integra* Thund. [53]. The implication is that this strategy makes the insect’s reproductive success dependent on the plant’s fecundity, i.e. pollination and fertilization. Insect success further depends on the ability of ovipositing females to detect such fertilized ovules. In the case of parasitism of pre-fertilization seed, reproductive success of the parasites is not linked to plant fecundity because, consistently with the seminal work on *M*. *spermotrophus* and Douglas fir [19], insects that adopt this strategy probably have the ability to prevent abortion in the event of pollination or fertilization failure. We speculate a link of such fitness implications with the successful radiation of the pre-fertilization *Megastigmus* species from an ancestral post-fertilization clade over evolutionary time scales [27]. Species specialized on the Pinaceae indeed account for a third of the total species diversity in the phytophagous group of the *Megastigmus* genus [29].

If this study shows how plant reproductive phenology can be a key factor in the evolution of host specialization in insect parasites, one important applied outcome is that a pre- fertilization parasitism strategy may favour invasiveness in such pest forest insects. Distribution patterns and abundance of univoltine and short-lived herbivores are expected to be noticeably affected by disrupting factors of synchrony between their life history and host plant phenology such as changes in climate or distribution range [52,54]. This may have a more critical meaning for specialists, for which the possibility of refugia on alternative hosts is limited. In a context of increasing worldwide unregulated seed trades, trans-continental successful invasions of seed wasps have been abundantly reported in pinaceous ecosystems, while they are surprisingly lacking in cupressaceous ones [26,55,56]. Targeting host reserves accumulated before fertilization may be more advantageous than targeting those accumulated after fertilization. There may be three reasons. First, independence from the presence of a fertilized host at emergence counterbalances the possibility of host-parasite phenological asynchrony in the new environment [54]. Second, independence from a host’s fecundity limits the parasite’s susceptibility to spatio-temporal variation in suitable resources in which to oviposit, e.g. in years (or areas) when pollen production or pollination-fertilization success is low. Finally, because the benefits of ovipositing should be maximized per unit time spent foraging while minimizing the costs [6], the possibility of targeting any ovule in any cone and of preventing seed abortion may efficiently reduce the amount of time required to find those that are suitable for larval development and ensure a sustainable environment for larval growth. Consequently, we posit that developing a pre-fertilization targeting strategy probably makes better parasitic invaders, and pinaceous ecosystems may be more vulnerable to seed-specialist invasions than cupressaceous ones. This hypothesis is supported by the results of a recent survey that shows very low rates of Megastigmus parasitism in New World cupressaceous species compared to pinaceous [57].

## Supporting Information

S1 Dataset Raw emergence data of cedar and cypress seed wasps used in this study. (XLSX)
